# Multiplex Recombinase Polymerase Amplification Assay for the Simultaneous Detection of Three Foodborne Pathogens in Seafood

**DOI:** 10.3390/foods9030278

**Published:** 2020-03-03

**Authors:** Biao Ma, Jiali Li, Kai Chen, Xiaoping Yu, Chuanxin Sun, Mingzhou Zhang

**Affiliations:** 1 Zhejiang Provincial Key Laboratory of Biometrology and Inspection & Quarantine, China Jiliang University, Hangzhou 310018, China; 16a0701109@cjlu.edu.cn (B.M.); s1709071012@cjlu.edu.cn (J.L.); s1809071005@cjlu.edu.cn (K.C.); yxp@cjlu.edu.cn (X.Y.); 2 Department of Plant Biology, Uppsala BioCenter, Linnean Centre for Plant Biology, Swedish University of Agricultural Science (SLU), SE-75007 Uppsala, Sweden; Chuanxin.Sun@slu.se

**Keywords:** recombinase polymerase amplification, lateral flow dipstick, multiplex detection, quantitative detection, *Staphylococcus aureus*, *Vibrio parahaemolyticus*, *Salmonella* Enteritidis

## Abstract

Foodborne pathogens can cause foodborne illness. In reality, one food sample may carry more than one pathogen. A rapid, sensitive, and multiple target method for bacteria detection is crucial in food safety. For the simultaneous detection of *Staphylococcus aureus*, *Vibrio parahaemolyticus*, and *Salmonella* Enteritidis, multi-objective recombinase polymerase amplification (RPA) combined with a lateral flow dipstick (LFD) was developed in this study. The whole process, including amplification and reading, can be completed in 15 min at 37 °C. The detection limits were 2.6 × 10^1^ CFU/mL for *Staphylococcus aureus*, 7.6 × 10^1^ CFU/mL for *Vibrio parahaemolyticus*, and 1.29 × 10^1^ CFU/mL for *Salmonella* Enteritidis. Moreover, colored signal intensities on test lines were measured by a test strip reader to achieve quantitative detection for *Staphylococcus aureus* (R^2^ = 0.9903), *Vibrio parahaemolyticus* (R^2^ = 0.9928), and *Salmonella* Enteritidis (R^2^ = 0.9945). In addition, the method demonstrated good recoveries (92.00%–107.95%) in the testing of spiked food samples. Therefore, the multiplex LFD-RPA assay is a feasible method for the rapid, sensitive, and quantitative detection of bacterial pathogens in seafood.

## 1. Introduction

Seafood products have become increasingly popular among consumers across the world. However, seafood has frequently been associated with foodborne illness because it is easily contaminated with pathogens during cultivation, handling, and processing. Foodborne pathogens seriously threaten human health and can cause various diseases and even death. The World Health Organization released a report in 2015 stating that 70% of foodborne diseases were caused by pathogenic microorganisms. The five most prevalent foodborne pathogens are *Staphylococcus aureus*, *Vibrio parahaemolyticus*, *Salmonella*, *Listeria monocytogenes,* and *Escherichia coli O157:H7* [1]. Among them, *Staphylococcus aureus*, *Vibrio parahaemolyticus*, and *Salmonella* are important foodborne pathogens in seafood products [2,3]. *Staphylococcus aureus* has been frequently reported as a pathogenic bacterium associated with food poisoning worldwide [4]. Eating food contaminated with *Staphylococcus aureus* can cause food poisoning and even life-threatening diseases [5]. *Vibrio parahaemolyticus* is an important foodborne pathogen that might give rise to acute gastroenteritis with nausea, headache, and a low fever [6,7]. *Salmonella* is a major foodborne pathogen, and it can cause an illness called salmonellosis [8]. The consumption of food contaminated with *Salmonella* might lead to typhoid fever, septicemia, gastroenteritis, and even death [9,10]. Since these three pathogens might coexist in one sample with a relatively low concentration, it is crucial to develop a rapid, highly sensitive, and accurate detection method for the simultaneous identification of multiple targets.

The culture-based method is used as the gold standard for the detection of pathogens, even though it takes more than two days [11]. With the development of biotechnology, many novel techniques have been applied in the detection of pathogenic bacteria. Among them, the detection techniques of molecular biology diagnosis have the advantages of rapid amplification, a high sensitivity, and a strong specificity [12]. However, most molecular tests require complicated instruments and professional training. For instance, the polymerase chain reaction (PCR) has been considered as a rapid and accurate method for the detection of foodborne pathogens. It can amplify a low concentration of target DNA in a few hours [13]. In addition, multiplex PCR has also been used in the detection of real food samples [14,15]. Nevertheless, methods based on PCR are not suitable for wide application in the field due to the dependence on thermal cycling instruments.

In order to simplify the experimental steps and reduce the dependence on the instrument, various isothermal amplifications of nucleic acids have been developed. Recombinase polymerase amplification (RPA) is one of the representative isothermal amplification techniques for the detection of nucleic acid [16]. The assay uses a recombinase enzyme to facilitate the binding of oligonucleotide primers to their complementary sequences of double-stranded DNA molecules. The process is aided by the single-stranded DNA binding protein, which prevents dissociation of the primers. The replication is performed by a DNA polymerase, which has the strand-displacement activity necessary to synthesize a new complementary DNA strand [17]. The amplified products can be detected after being incubated at 37–42 °C in 5–20 min [18]. Accurate and simple readouts are important for the analysis of amplification products. The gel electrophoresis, fluorescence labeling, and lateral flow immunoassays have served for the analysis of RPA amplicons [19]. The lateral flow dipstick (LFD) is a simple, fast, and visual immune assay for amplified product detection. The testing is achieved by binding to a tag-specific antibody at the detection line and then cross-linking it with a second tag-specific antibody on colloid gold nanoparticles [20,21]. After 5–10 min of incubation, results can be observed by colored signals in a control line and test line. They can be semi-quantitatively analyzed and are directly observable by the naked eye. The LFD-RPA assay has been applied for a number of pathogens, including *Mycobacterium avium* [22], *Agrobacterium* spp. [23], *Listeria monocytogenes* [24], and *Streptococcus pneumoniae* [25]. However, previous studies have focused on single targets. It is challenging for multiple detection to avoid the cross-reaction between several specific antibodies in the dipstick.

In the present study, we report a combination of isothermal RPA and LFD for the simultaneous detection of *Staphylococcus aureus*, *Vibrio parahaemolyticus*, and *Salmonella* Enteritidis. To solve the bottleneck of an accurate readout, a test strip reader (TSR-200, Allsheng Instruments Co. Ltd., Hangzhou, China) was employed to scan and evaluate the colored signals on the lateral flow dipsticks. After determining the analytical sensitivity and specificity, the approach was successfully applied in the detection of actual food samples. In general, the multiplex LFD-RPA assay is a simple, visual, specific, and measurable technique, and it will be helpful for diagnostics of foodborne pathogens in the field.

## 2. Materials and Methods

### 2.1. Bacterial Strains and DNA Template Preparation

A total of 27 bacterial strains, including 4 *Staphylococcus aureus* strains, 4 *Vibrio parahaemolyticus* strains, 4 *Salmonella enterica* strains, and 15 other foodborne strains, were used to determine the specificity of the multiplex LFD-RPA assay in the study (Table 1). *Staphylococcus aureus* (ATCC25923), *Vibrio parahaemolyticus* (ATCC17802), and *Salmonella* Enteritidis (ATCC13076) were employed as reference strains to optimize the reaction system and sensitivity analysis of multiplex LFD-RPA. *Vibrio parahaemolyticus* strains were cultured in alkaline peptone water (APW, Hopebio, Qingdao, China) supplemented with 3% NaCl at 37 °C for 18 h, and other strains were cultured in Luria–Bertani broth (LB, Sangon, Shanghai, China) at 37 °C for 18 h. The bacterial culture was used for the extraction of nucleic acids or conventional plate counting. To determine the sensitivity, *Vibrio parahaemolyticus* (ATCC17802) was grown to the mid-exponential growth phase and serially diluted 10-fold in APW medium. *Staphylococcus aureus* (ATCC25923) and *Salmonella* Enteritidis (ATCC13076) were serially diluted 10-fold in LB medium. The initial cell number was quantified by plate counting on plate count agar (Hopebio, Qingdao, China), according to the bacteriological analytical manual (BAM) [26].

The total DNA of the pure culture was extracted by using lysis buffer. The lysis buffer contained 200 mM Guanidine hydrochloride, 50 mM Tris-HCl (pH 8.8), 0.01% sodium dodecyl sulfate (SDS), and 100 mM NaCl. The bacterial solution (1 mL) was centrifuged for 1 minute at 6000 rpm. The pellets were recovered and mixed with the lysis buffer (200 μL). After another centrifugation at 6000 rpm for 1 minute, the supernatant was stored at −80 °C until use. The DNA concentration was measured by a spectrophotometer (DU730, Beckman Coulter, Burea, CA, USA).

### 2.2. Design of the RPA Primers

Based on the *nuc* gene (Genebank accession: EF529607.1) of *Staphylococcus aureus*, *toxR* gene (Genebank accession: GQ228073.1) of *Vibrio parahaemolyticus*, and *fimY* gene (Genebank accession: JQ665438.1) of *Salmonella enterica*, three sets of RPA primers were designed by using the software Primer Premier 5.0 (Premier Biosoft, San Francisco, CA, USA). The primers (Table 2) with target-specific labels were tagged with biotin and digoxin (primers for the *nuc* gene), carboxy fluorescein (FAM), and digoxin (primers for the *toxR* gene) or Cyanine 5 (Cy5) and digoxin (primers for the *fimY* gene). All primers were synthesized by Invitrogen Biotechnology Co. Ltd (Shanghai, China).

### 2.3. Multiplex RPA Reactions in Solution

RPA was performed according to the manufacturer’s instructions with a TwistAmp Basic Kit (TwistDX, Cambridge, UK). Briefly, the reaction mixtures contained 25 μL 2× reaction buffer; 11.5 μL ddH_2_O; 2 μL each of *Staphylococcus aureus*, *Vibrio parahaemolyticus*, and *Salmonella* Enteritidis specific primer (10 μM); enzymes; 0.5 μL of each template; and 2.5 μL Magnesium acetate. The amplification reaction proceeded at 37 °C for 20 min. Finally, the lateral flow immunoassay was employed to visualize RPA amplifications.

### 2.4. Visualization and Quantification of Lateral Flow Dipsticks

**Figure 1:**
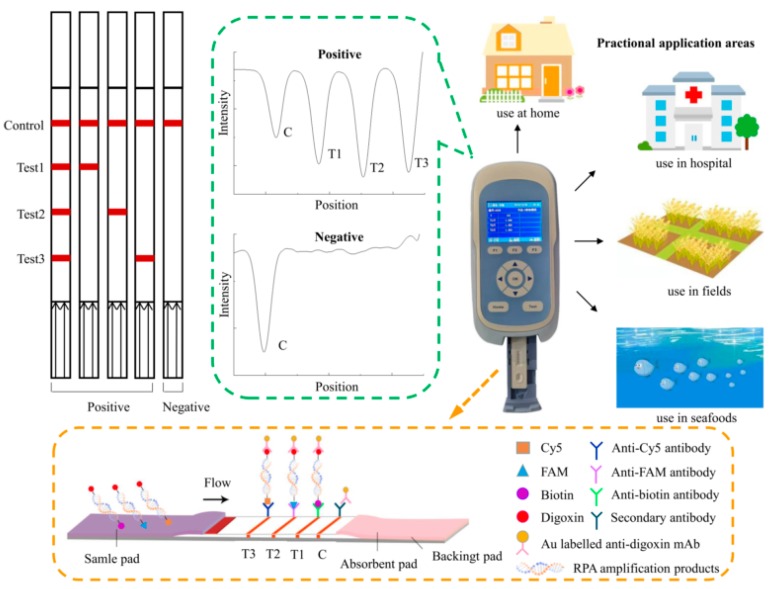
The working principle and potential application of the triplex recombinase polymerase amplification combined with a lateral flow dipstick (LFD-RPA) assay.

The lateral flow dipstick was mainly composed of a sample pad, a conjugate pad, an absorbent pad, a backing card, and a nitrocellulose filter (NC) membrane with three test lines and one control line (Figure 1). The membrane was HF180 nitrocellulose, and the capillary flow rate was 180 s/4 cm. Colloidal gold labeled with anti-digoxin monoclonal antibody was sprayed on the conjugate pad. The three test lines were prepared separately with 0.65 mg/mL anti-biotin monoclonal antibody, 0.325 mg/mL anti-FAM monoclonal antibody, and 0.3 mg/mL anti-Cy5 monoclonal antibody. Immobilized goat anti-mouse polyclonal antibody (pAb) on the control line served as an assay control. The NC membrane was dried at 37 °C for 12 h. The assembled strips were cut to a 2.5 mm width and stored in a desiccator at room temperature until use.

Before being pipetted onto the test strip, the amplification products were diluted 50 times by a running buffer, which contained PBS and 3% Tween 20. A strip test reader was used to scan the strips and evaluate the intensity of the bands on the test lines.

### 2.5. RPA-LFD Assay

The principle of the multiplex LFD-RPA assay is shown in Figure 1. Firstly, multiplex RPA amplification was performed to generate amplification products of biotin-digoxin-, FAM-digoxin-, and Cy5-digoxin-tagged double-stranded DNA by using the labeled upstream primer and downstream primer. Then, the amplification solution was loaded onto the sample pad and diverted to the other end of the strip by capillary force. The labeled duplex DNA was combined with the anti-digoxin monoclonal antibody colloidal gold on the conjugate pad. Then, as the amplification solution continued migrating, the biotin-, FAM-, and Cy5-labeled duplex DNA was captured by the three test lines, respectively, where were contained with a corresponding anti-biotin monoclonal antibody, anti-FAM monoclonal antibody, and anti-Cy5 monoclonal antibody. The uncaptured gold particles flowed through. They were immobilized by the antibody on the control line. In the absence of the target DNA, no red band was observed on the test line. Reddish bands were generated on the test lines and control line due to the accumulation of colloidal gold if the amplification was successfully generated. The entire procedure took approximately 5–10 min. The intensity of the test lines and control line was scanned by the test strip reader. The reader converted the received light signals from the test (T) lines and control (C) line into electrical signals with a “T value” and “C value”, and the results are described as the ratio of the T value to C value (T/C value).

### 2.6. Optimization of the LFD-RPA Conditions

In order to establish the multiplex RPA assay, initial experiments were undertaken in a single tube to screen primer ratios and test different reaction conditions. The simultaneous amplification system for *Staphylococcus aureus, Vibrio parahaemolyticus*, and *Salmonella* Enteritidis was optimized. In the experiments, the amplification efficiency of different targets was inconsistent. To achieve a similar amplification efficiency for the three fragments, the possibility of balancing primer ratios was explored. Five primer ratios were tested to determine the optimal primer ratios (150 nM primer concentration of *Vibrio parahaemolyticus*; five gradients of 150, 200, 300, 400, and 500 nM primer concentration of *Staphylococcus aureus* and *Salmonella* Enteritidis). On the basis of primer optimization, the optimal duration of time, the reaction temperature for multiplex RPA, and the salt concentrations of magnesium ions were examined. The temperature experiment was performed with various temperatures ranging from 30 to 50 °C, according to the above-mentioned protocol. Ten distinct reaction times were compared (2.5, 5, 7.5, 10, 12.5, 15, 17.5, 20, 22.5, and 25 min). In addition, different concentrations of magnesium ions were applied, which ranged from 0 to 16.8 mM. To determine the optimal conditions, the genomic DNA of *Staphylococcus aureus*, *Vibrio parahaemolyticus*, and *Salmonella* Enteritidis bacterial cultures (10^7^ CFU/mL) was extracted as the target template. All optimization experiments were performed in a metal bath (MiniT-100H, Allsheng Instruments Co. Ltd., Hangzhou, China).

### 2.7. Specificity and Sensitivity of Multiplex LFD-RPA

The genomic DNA (10^7^ CFU/mL) of 27 bacterial strains listed in Table 1 was extracted and added into the reaction to determine the specificity of the multiplex LFD-RPA assay.

In the sensitivity experiment, ten-fold serial dilutions of the three reference strains were used to confirm the detection limit. The genomic DNA was extracted from *Staphylococcus aureus*, *Vibrio parahaemolyticus*, and *Salmonella* Enteritidis bacterial cultures at concentrations ranging from 10^7^ to 10^0^ CFU/mL. Each concentration level was replicated three times, and each strip was scanned by the test strip reader three times.

### 2.8. Application of Multiplex LFD-RPA in Artificially Contaminated Food Samples

*Staphylococcus aureus* and *Salmonella* Enteritidis were individually cultured in LB for 18 h at 37 °C. *Vibrio parahaemolyticus* was cultured in APW with 3% Nacl for 18 h at 37 °C. The cultures were used to prepare various concentrations of *Staphylococcus aureus*, *Vibrio parahaemolyticus*, and *Salmonella* Enteritidis. Sleevefish, shrimp, and cod samples were purchased from a local market (Hangzhou, China). All were confirmed to be negative for *Staphylococcus aureus*, *Vibrio parahaemolyticus*, and *Salmonella* Enteritidis, according to the BAM formulated by FDA [26]. Each sample (25.0 g ± 0.1 g) was mixed with 225 mL LB and homogenized under sterile conditions. Each sample’s homogenates were contaminated with 10^4^, 10^3^, 10^2^, or 10^1^ CFU/mL of *Staphylococcus aureus*, *Vibrio parahaemolyticus*, and *Salmonella* Enteritidis. Subsequently, each inoculated sample was collected for DNA extraction.

For the extraction of DNA from spiked samples and real food samples, 1 mL of each inoculated sample was collected for DNA extraction, and 10 mg/mL lysozyme (Bioteke Corporation, Beijing, China) was added at 37 °C for 5 min, followed by 20 mg/mL proteinase K (Bioteke Corporation, China) at 60 °C for 15 min, to digest proteins in the sample and break the bacterial cell wall. The genomic DNA was obtained using a bacterial genomic DNA extraction kit (Bioteke Corporation, China), according to the manufacturer’s protocol. Each extracted DNA sample was used in a multiplex RPA reaction. The inoculated samples were also tested based on the BAM. Non-inoculated samples were used as the negative control.

### 2.9. Field Sample Testing

Field samples were categorized into two groups based on the source: one group was purchased randomly from the local market, while the others were afforded by the Zhoushan Entry-Exit Inspection and Quarantine Bureau. Each sample (25.0 g ± 0.1 g) was added to 225 mL of LB and mixed well by swirling under sterile conditions. Then, it was enriched at 37 °C with shaking at 200 rpm for 16 h after being fully homogenized. The DNA was extracted as described in Section 2.8. All samples were detected by multiplex LFD-RPA and BAM assays.

### 2.10. Data Analysis

The results of LFD were read by a TSR-200 Test Strip Reader. The standard curve of the LFD-RPA assay was plotted according to the T/C value and logarithm of the bacterial culture concentration. The average was used to calculate the recovery rate (the ratio of the actual measured concentration to the artificial contaminated concentration). Data collected from the LFD-RPA assay, including standard curves for quantifying *Staphylococcus aureus*, *Vibrio parahaemolyticus*, and *Salmonella* Enteritidis cells, were analyzed using TSR-200 reader software (Allsheng Instruments Co. Ltd., Hangzhou, China) and Microsoft Excel software (Microsoft Inc., Washington, DC, USA).

## 3. Results

### 3.1. Establishment and Optimization of the Multiplex LFD-RPA Assay

**Figure 2:**
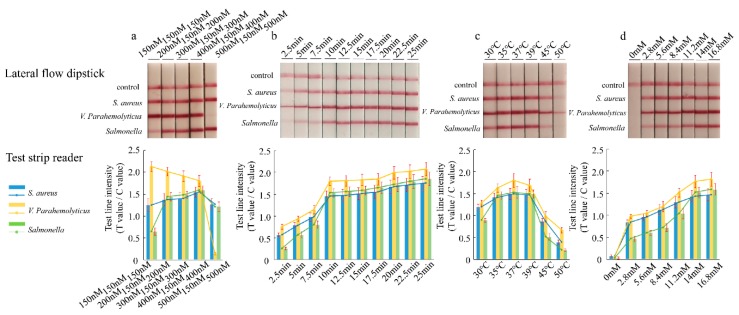
Optimization of the recombinase polymerase amplification reaction. Ratios of primers (**a**), incubation time (**b**), temperature (**c**), and the concentration of magnesium ions (**d**) using target DNA. LFD test results (top) and LFD test line quantification (bottom). The test line intensity was the ratio of the reflected light for the test line and control line.

Primers for the *Vibrio parahaemolyticus* fragment were decreased to 150 nM, and a range of concentrations of the primers for the *Staphylococcus aureus* and *Salmonella* Enteritidis fragments (150–500 nM) were evaluated. The optimization experiments were conducted at 37 °C, with an incubation time of 20 min. After that, the amplification products were analyzed by using a lateral flow immunoassay. In terms of the equivalent amplification efficiency, the results were obtained when using 150 nM *Vibrio parahaemolyticus*, 400 nM *Staphylococcus aureus*, and 400 nM *Salmonella* Enteritidis primers (Figure 2a). The reaction times of 2.5, 5, 7.5, 10, 12.5, 15, 17.5, 20, 22.5, and 25 min were evaluated. The signal on the lateral flow dipstick could be detected after 5 min at 37 °C. The intensity of the band increased with longer reaction times (Figure 2b). The band was brighter when the amplification time was 20 or 25 min, but the reaction time was longer. As a result, 10 min was the appropriate reaction time. The RPA assay was conducted under isothermal conditions between 30 and 50 °C. According to the results of strips and the test strip reader, the best reaction temperature of the LFD-RPA assay was 37 °C (Figure 2c). Subsequently, seven magnesium ion concentration gradients of 0, 2.8, 5.6, 8.4, 11.2, 14, and 16.8 mM were tested. As shown in Figure 2d, there were no obvious differences between the magnesium ion concentrations from 14 to 16.8 mM. Therefore, 14 mM magnesium acetate was found to provide an optimal performance for the LFD-RPA assay.

### 3.2. Sensitivity and Specificity of the Multiplex LFD-RPA Assay

**Figure 3:**
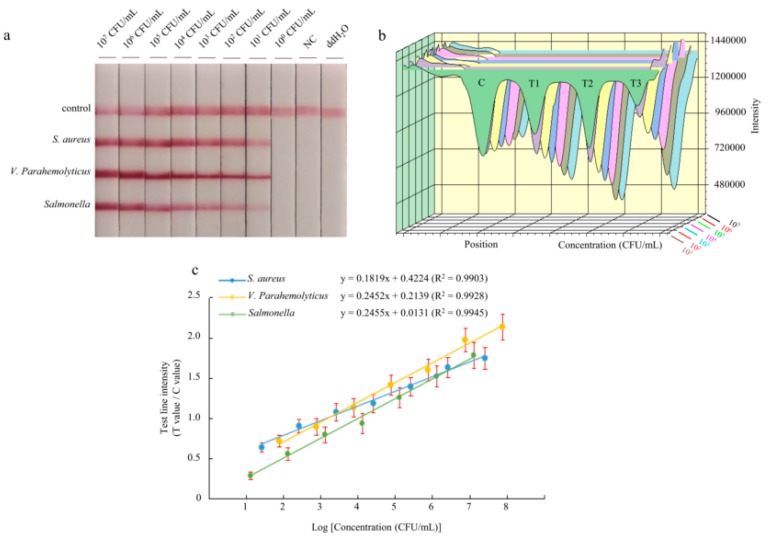
Reaction sensitivity of triplex LFD-RPA for the *Staphylococcus aureus* strain culture, *Vibrio parahaemolyticus* strain culture, and *Salmonella* Enteritidis strain culture. The amplified products could be observed by the naked eye by using a lateral flow dipstick (**a**). The intensity (**b**) was used for quantitative analysis, and it shows a linear correlation (**c**) with the concentration of pure cultures. The test line intensity was the ratio of the reflected light for the test line and control line.

To assess the sensitivity of the multiplex LFD-RPA assay, 10-fold serial dilutions of pure bacteria solution were prepared, and the purified bacterial genomic DNA was used as a template. The amplification products were analyzed by a lateral flow immunoassay. The color of the test lines was not visible to the naked eye when bacterial concentrations were below 10^1^ CFU/mL for *Staphylococcus aureus*, *Vibrio parahaemolyticus*, and *Salmonella* Enteritidis (Figure 3a). Therefore, the visual detection limits of multiplex LFD-RPA for the simultaneous detection of *Staphylococcus aureus*, *Vibrio parahaemolyticus*, and *Salmonella* Enteritidis were 2.6 × 10^1^, 7.6 × 10^1^, and 1.29 × 10^1^ CFU/mL, respectively. Using the strip test reader to detect the intensity of the colored signal on the test lines, it is possible to achieve quantitative results. The colored signal intensity increased with a high concentration of DNA template (Figure 3b). The standard linear curves have correlation coefficients of determination for *Staphylococcus aureus* (*R*^2^ = 0.9903), *Vibrio parahaemolyticus* (*R*^2^ = 0.9928), and *Salmonella* Enteritidis (*R*^2^ = 0.9945) (Figure 3c). There were significant correlations between the detection threshold and the template concentration. Moreover, the detection limit of the reader was 2.6 × 10^1^ CFU/mL for *Staphylococcus aureus*, 7.6 × 10^1^ CFU/mL for *Vibrio parahaemolyticus*, and 1.29 × 10^1^ CFU/mL for *Salmonella* Enteritidis.

**Figure 4:**
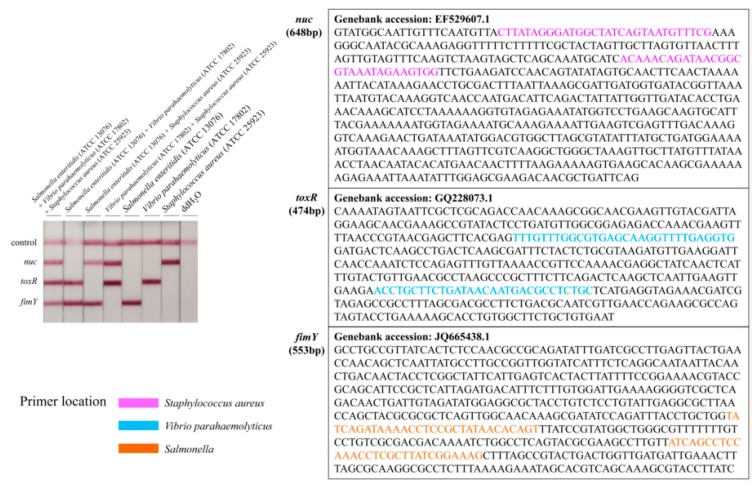
Specificity between the *nuc* gene for *Staphylococcus aureus*, *toxR* gene for *Vibrio parahaemolyticus*, and *fimY* gene for *Salmonella enterica* and the primer location in genes.

To determine the specificity of the primers, all of the 27 bacterial strains were investigated in LFD-RPA experiments, including 4 *Staphylococcus aureus* strains, 4 *Vibrio parahaemolyticus* strains, 4 *Salmonella enterica* strains, and 15 other foodborne strains. Specific experimental results showed that only *Staphylococcus aureus*, *Vibrio parahaemolyticus*, and *Salmonella* Enteritidis strains had positive results, while the other strains had negative results (Table 1). The specificity results also indicated that there was no cross-reactivity among the *nuc* gene for *Staphylococcus aureus*, *toxR* gene for *Vibrio parahaemolyticus*, and *fimY* gene for *Salmonella enterica* (Figure 4).

### 3.3. Application of the Multiplex LFD-RPA Assay in Food Samples

Fresh sleevefish, shrimp, and cod samples were collected for *Staphylococcus aureus, Vibrio parahaemolyticus*, and *Salmonella* Enteritidis detection to demonstrate the application of the multiplex LFD-RPA assay. The samples were spiked with different concentrations of *Staphylococcus aureus*, *Vibrio parahaemolyticus*, and *Salmonella* Enteritidis. The genomic DNA of each tested sample was extracted for LFD-RPA detection. Meanwhile, inoculated samples were also tested according to the BAM. Table 3 shows that the quantitative detection results of spiked food for *Staphylococcus aureus*, *Vibrio parahaemolyticus*, and *Salmonella* Enteritidis by the multiplex LFD-RPA assay were similar to those obtained with a pure bacterial solution. As shown in Table 3, the recoveries of *Staphylococcus aureus* in fresh sleevefish, shrimp, and cod were 92%–106.22%; the recoveries of *Vibrio parahaemolyticus* in fresh sleevefish, shrimp, and cod were 93.73%–107.59%; and the recoveries of *Salmonella* Enteritidis in fresh sleevefish, shrimp, and cod were 92.96%–106.66%. The detection limits of the LFD-RPA assay for *Staphylococcus aureus*, *Vibrio parahaemolyticus*, and *Salmonella* Enteritidis were 4.5 × 10^1^, 8.3 × 10^1^, and 2.7 × 10^1^ CFU/mL, respectively.

### 3.4. Detection of Field Samples

For field sample testing, 123 samples belonging to eight types were analyzed (Table 4). *Staphylococcus aureus*, *Vibrio parahaemolyticus*, and *Salmonella* Enteritidis were tested in all samples using multiplex LFD-RPA and BAM assays. The positive detection rates for *Staphylococcus aureus* of samples from the local market and the Zhoushan Entry-Exit Inspection and Quarantine Bureau were both 0%. The positive detection rates for *Vibrio parahaemolyticus* and *Salmonella* Enteritidis of samples from the local market were both 1.9%, and the positive detection rates of samples from the Zhoushan Entry-Exit Inspection and Quarantine Bureau were both 0%.

## 4. Discussion

*Staphylococcus aureus*, *Vibrio parahaemolyticus*, and *Salmonella* are common foodborne pathogens that can cause various foodborne illnesses, such as typhoid fever, septicemia, and gastroenteritis, and even death. Therefore, the rapid, sensitive, and reliable detection of *Staphylococcus aureus*, *Vibrio parahaemolyticus*, and *Salmonella* Enteritidis is essential in reducing the risk factor caused by the three pathogens. Numerous methods have been established to detect pathogenic bacteria in food, such as real-time PCR [27], enzyme-linked immunosorbent assays [28], and electrochemical biosensors [29]. However, these approaches are not suitable for wide application in the field due to the need for specific equipment and the requirement of time-consuming procedures. On this basis, a number of isothermal amplification methods have been developed, including nucleic acid sequence-based amplification (NASBA) [30], Helicase-dependent isothermal DNA amplification (HDA) [31], and loop-mediated isothermal amplification (LAMP) [32].

Nevertheless, since multiple pathogens often coexist in food, traditional single target nucleic acid detection can no longer meet the needs of multiple detection. Multiple nucleic acid detection technology can achieve the simultaneous and rapid amplification of multiple pathogens in a single reaction system [33]. This method not only retains the specificity and sensitivity of the detection, but also reduces the number of operating steps and reagents. Multiplex PCR and multiplex LAMP are two widely used multiplex nucleic acid detection technologies. Multiplex PCR and multiplex LAMP have been applied to the detection of a variety of foodborne pathogens, for example, in the simultaneous detection of *Pasteurella multocida*, *Salmonella enterica*, *Riemerella anatipestifer*, and *Escherichia coli* in ducks by using multiplex PCR [34], and the triplex detection for three genes of methicillin-resistant *Staphylococcus aureus* (MRSA) by using multiplex LAMP [35]. However, instrument dependence and thermal cycling have limited the application in the field of PCR. In addition, the amplification efficiency of crude samples has much room for improvement for use in the field [36]. Meanwhile, RPA shows significant advantages, such as a short incubation time, lower incubation temperatures, and a high tolerance to sample impurities. It is possible to perform multiple RPA amplifications in one reaction system, and this has been successfully reported, for example, in a real-time fluorescent duplex RPA assay for the detection of *Campylobacter coli* and *jejuni* from eggs and chicken products [37], a duplex SRES-based lateral flow assay for the detection of *Listeria monocytogenes* and *Salmonella* Enteritidis [1], and a duplex lateral flow assay for the detection of P-35S and T-nos in genetically modified organisms [38]. However, there have been few reports on the LFD-RPA assay for the simultaneous detection of three or more foodborne pathogens [39]. In this study, we have described a specific and quantitative multiplex LFD-RPA method for the accurate identification of the three foodborne pathogens simultaneously. The multiplex LFD-RPA assay was operated at a constant temperature without a specialized instrument. The presented LFD-RPA method potentiates highly accessible and sensitive nucleic acid amplification outside of the laboratory.

For multiple RPA reactions, the chosen target sequences and the design of primers are the intrinsic determinants for the amplification efficiency [39]. The *nuc* gene of *Staphylococcus aureus*, *toxR* gene of *Vibrio parahaemolyticus*, and *fimY* gene of *Salmonella enterica* were used to design RPA primers. The *nuc* gene, which is involved in regulating thermostable nuclease, is unique to *Staphylococcus aureus* and is used as an indicator of *Staphylococcus aureus* contamination [2]. The *toxR* gene, with a low degree of homology between different species, had a high accuracy in identifying *Vibrio parahaemolyticus* [40]. The *fimY* gene is involved in regulating the type 1 fimbrial expression of *Salmonella enterica*. The amino acid sequence of *fimY* shares very little homology with other known prokaryotic proteins in the GenBank database [41]. The lengths of RPA primers are relatively long, with a recommended minimum of 30 nucleotides, but typical length of between 32 and 35 nucleotides [42]. However, in multiple RPA reactions, some targets are amplified using RPA much more rapidly and efficiently than others [43]. We adjusted the primer concentration to 150 nM for *Vibrio parahaemolyticus* primers and 400 nM for *Staphylococcus aureus* and *Salmonella* Enteritidis primers, to obtain an equivalent amplification efficiency of the three fragments. The reaction temperature is one of the most important contributing extrinsic factors that allows the RPA assay to achieve an optimal efficiency and analytical sensitivity. The recommended RPA reaction temperature is between 37 and 42 °C. The experiments could be executed in a water bath or heating block. Several research studies have indicated that the RPA reaction can be performed at an ambient temperature, even body temperature [44,45]. The temperature range was between 30 and 50 °C in our experiments, and 37 °C was chosen as the best reaction temperature. The optimal conditions for the multiplex detection of *Staphylococcus aureus*, *Vibrio parahaemolyticus*, and *Salmonella* Enteritidis by the RPA assay were determined to be 37 °C for 10 min in this study.

Recently, a number of different methods have been coupled with RPA, including the flocculation assay [46,47], silicon microring resonator (SMR)-based photonic detection [48], and surface-enhanced Raman scattering (SERS) [1]. Compared with those detection methods, the lateral flow dipstick is a faster and simpler method. The amplification products were directly detected using the lateral flow dipstick, and the resulting visualization could be obtained within 5 min. To achieve triple detection, each of the upstream primers was labeled with biotin, FAM, and Cy5 at the 5′ end. All of the downstream primers were labeled with digoxin at the 5′ end. Three double-labeled detectable products were formed in amplification. Meanwhile, the lateral flow dipsticks with three test lines were prepared for RPA product detection. However, LFD is generally considered as a qualitative detection method due to the lack of results analysis apparatus. In this study, a test strip reader was used to analyze the results of the dipsticks. The reader (TSR-200) could scan the test strips and detect the intensity of the reflected light by using modern photoelectric technology. The accurate intensity data of the colored signal on the test lines can be read and obtained. Such a reader can decrease the man-made influence and increase the correctness of diagnosis. The LFD-RPA assay became a more sensitive and quantitative detection method of pathogenic bacteria when being combined with the test strip reader. This assay can simultaneously detect as few as 2.6 × 10^1^ CFU/mL for *Staphylococcus aureus*, 7.6 × 10^1^ CFU/mL for *Vibrio parahaemolyticus*, and 1.29 × 10^1^ CFU/mL for *Salmonella* Enteritidis. Furthermore, the sensitivity showed that there were significant correlations between the colored signals and the template concentration of *Staphylococcus aureus*, *Vibrio parahaemolyticus*, and *Salmonella* Enteritidis (*R*^2^ = 0.9903, *R*^2^ = 0.9928, and *R*^2^ = 0.9945, respectively). In addition, there was not only no cross-amplification between the three target bacteria, but also no cross-amplification with other bacteria. The results indicate that the established method was highly specific. In order to more comprehensively confirm the validity and applicability of the sample analysis by using multiplex LFD-RPA, the recovery of spiked food samples was analyzed, and it was also compared with microbial culturing. The results suggested that the LFD-RPA assay had higher recovery rates in the spiked food samples. Compared with the published triplex LFD-RPA detection assay [49], this study could avoid naked-eye reading mistakes by using the test strip reader. In conclusion, the multiplex LFD-RPA assay is a rapid, high-sensitivity, and high-throughput detection method for foodborne pathogens.

## 5. Conclusions

In this study, a multiplex LFD-RPA assay was developed for the rapid detection of foodborne pathogens in seafood. The assay could simultaneously detect *Staphylococcus aureus*, *Vibrio parahaemolyticus*, and *Salmonella* Enteritidis, and the test strip reader showed the quantification result. The multiplex LFD-RPA assay is time-saving, simple, sensitive, and specific, allowing a visual and measurable analysis of three fragments in a single reaction. Moreover, the assay has potential application in the field, since the amplification could be completed in 10 min at 37 °C and could be observed by the naked eye. The application of the test strip reader decreased the man-made influence and allowed quantification detection. It improved the correctness of diagnosis. In summary, the multiplex LFD-RPA has practical significance for the rapid detection of foodborne pathogens in the field.

## Figures and Tables

**Table 1:** Information of bacterial strains used for specificity tests.

Species	ID of Strains	Multiple LFD-RPA Results		
		*nuc*	*toxR*	*fimY*
*Staphylococcus aureus*	ATCC 25923	+	-	-
*Staphylococcus aureus*	GIMCC 1.142	+	-	-
*Staphylococcus aureus*	CICC 10001	+	-	-
*Staphylococcus aureus*	CICC 21648	+	-	-
*Vibrio parahaemolyticus*	ATCC 17802	-	+	-
*Vibrio parahaemolyticus*	ATCC 33847	-	+	-
*Vibrio parahaemolyticus*	H4-3 *	-	+	-
*Vibrio parahaemolyticus*	FJ14A *	-	+	-
*Salmonella* Enteritidis	ATCC 13076	-	-	+
*Salmonella* Enteritidis	GIMCC 1.345	-	-	+
*Salmonella* Enteritidis	CMCC 50041	-	-	+
*Salmonella enterica* subsp. *enterica*	CICC 10982	-	-	+
*Legionella pneumophila*	ATCC 33152	-	-	-
*Legionella pneumophila*	07 *	-	-	-
*Vibrio cholera*	GIMCC 1.449	-	-	-
*Vibrio cholera*	007zs0902-2009^a^	-	-	-
*Escherichia coli* O157:H7	ATCC 35150	-	-	-
*Escherichia coli* O157:H7	61 *	-	-	-
*Shigella flexneri*	CICC 10865	-	-	-
*Shigella sonnei*	GIMCC 1.424	-	-	-
*Listeria monocytogenes*	ATCC 19115	-	-	-
*Listeria monocytogene*	CICC 21633	-	-	-
*Cronobacter Sakazakii*	GIMCC 1.296	-	-	-
*Cronobacter Sakazakii*	CS-3 *	-	-	-
*Campylobacter jejuni* subsp. *jejuni*	ATCC 33560	-	-	-
*Enterobacter aerogenes*	CICC 10293	-	-	-
*Yersinia enterocolitica*	ATCC 23715	-	-	-

GIMCC: Guangdong Microbiology Culture Center, Guangdong, China; CMCC: National Center for Medical Culture Collections, China; ATCC: American Type Culture Collection, Virginia, USA; CICC: China Center of Industrial Culture Collection, Shanghai, China; * Afforded by Zhoushan Entry-Exit Inspection and Quarantine Bureau, Zhejiang, China; +: positive result; -: negative result; LFD-RPA: recombinase polymerase amplification combined with a lateral flow dipstick.

**Table 2:** Sequences of *Staphylococcus aureus*, *Vibrio parahaemolyticus*, and *Salmonella enterica* recombinase polymerase amplification (RPA) primers.

Target Name	Primer Name	Sequence (5′–3′)	Fragment Length
*Staphylococcus aureus* (*nuc*)	Forward primer	5′-Biotin-CTTATAGGGATGGCTATCAGTAATGTTTCG-3′	158bp
	Reverse primer	5′-Digoxin-CCACTTCTATTTACGCCGTTATCTGTTTGT-3′	
*Vibrio parahaemolyticus* (*toxR*)	Forward primer	5′-FAM-TTTGTTTGGCGTGAGCAAGGTTTTGAGGTG-3′	230bp
	Reverse primer	5′-Digoxin-GCAGAGGCGTCATTGTTATCAGAAGCAGGT-3′	
*Salmonella enterica* (*fimY*)	Forward primer	5′-Cy5-TATCAGATAAAACCTCCGCTATAACACAGT-3′	133 bp
	Reverse primer	5′-Digoxin-CTTTCCGATAAGCGAGGTTTGGAGGCTGAT-3′	

**Table 3:** The recoveries of *Staphylococcus aureus*, *Vibrio parahaemolyticus* and *Salmonella* Enteritidis in spiked food samples by multiple LFD-RPA.

Sample (*n* = 6 Each)	*Staphylococcus aureus*				*Vibrio parahaemolyticus*				*Salmonella* Enteritidis			
	Inoculation Level (CFU/mL)	LFD-RPA Detected Concentration (CFU/mL)	Recovery (%)	BAM	Inoculation Level (CFU/mL)	LFD-RPA Detected Concentration (CFU/mL)	Recovery (%)	BAM	Inoculation Level (CFU/mL)	LFD-RPA Detected Concentration(CFU/mL)	Recovery (%)	BAM
Sleevefish	4.5 × 10^4^	4.29 × 10^4^	95.33	+	8.3 × 10^4^	8.93 × 10^4^	107.59	+	2.7 × 10^4^	2.59 × 10^4^	95.92	+
	4.5 × 10^3^	4.35 ×10^3^	96.66	+	8.3 × 10^3^	8.51 × 10^3^	102.53	+	2.7 × 10^3^	2.56 × 10^3^	94.81	+
	4.5 × 10^2^	4.66 × 10^2^	103.56	+	8.3 × 10^2^	8.13 × 10^2^	98.95	+	2.7 × 10^2^	2.61 × 10^2^	96.6	+
	4.5 × 10^1^	4.14 × 10^1^	92.00	-	8.3 × 10^1^	7.91 × 10^1^	95.30	-	2.7 × 10^1^	2.75 × 10^1^	101.85	-
Shrimp	4.5 × 10^4^	4.72 × 10^4^	104.89	+	8.3 × 10^4^	8.15 × 10^4^	98.19	+	2.7 × 10^4^	2.78 × 10^4^	102.96	+
	4.5 × 10^3^	4.42 × 10^3^	98.22	+	8.3 × 10^3^	8.67 × 10^3^	104.46	+	2.7 × 10^3^	2.53 × 10^3^	93.70	+
	4.5 × 10^2^	4.21 × 10^2^	93.56	+	8.3 × 10^2^	8.96 × 10^2^	107.95	+	2.7 × 10^2^	2.82 × 10^2^	104.44	+
	4.5 × 10^1^	4.15 × 10^1^	92.22	-	8.3 × 10^1^	7.96 × 10^1^	95.90	-	2.7 × 10^1^	2.56 × 10^1^	94.81	-
Cod	4.5 × 10^4^	4.78 × 10^4^	106.22	+	8.3 × 10^4^	8.72 × 10^4^	105.06	+	2.7 × 10^4^	2.84 × 10^4^	105.19	+
	4.5 × 10^3^	4.67 × 10^3^	103.78	+	8.3 × 10^3^	8.61 × 10^3^	103.73	+	2.7 × 10^3^	2.88 × 10^3^	106.66	+
	4.5 × 10^2^	4.36 × 10^2^	96.89	+	8.3 × 10^2^	8.06 × 10^2^	97.11	+	2.7 × 10^2^	2.67 × 10^2^	98.88	+
	4.5 × 10^1^	4.16 × 10^1^	92.44	-	8.3 × 10^1^	7.78 × 10^1^	93.73	-	2.7 × 10^1^	2.51 × 10^1^	92.96	-

BAM: bacteriological analytical manual.

**Table 4:** Detection of unknown samples from the market by a triplex assay compared with the culture method.

Resource	Samples	No. of Samples	Positive Number of Triplex LFD-RPA and BAM Method					
			*Staphylococcus aureus*		*Vibrio parahaemolyticus*		*Salmonella* Enteritidis	
			Triplex LFD-RPA	BAM	Triplex LFD-RPA	BAM	Triplex LFD-RPA	BAM
Local market	Shrimp	24	0	0	2	2	2	2
	Sleevefish	21	0	0	0	0	0	0
	*Trichiurus lepturus*	11	0	0	0	0	0	0
	Cod	15	0	0	0	0	0	0
	*Meretrix*	8	0	0	0	0	0	0
	Toasted *Muraenesox*	6	0	0	0	0	0	0
	Grilled fish	5	0	0	0	0	0	0
	Grilled yellow croaker	12	0	0	0	0	0	0
	Dried squid	4	0	0	0	0	0	0
	Total	106	0	0	2	2	2	2
	Positive detection rate (%)	/	0%	0%	1.9%	1.9%	1.9%	1.9%
Zhoushan Entry-Exit Inspection and Quarantine Bureau	Dried squid	17	0	0	0	0	0	0
	Positive detection rate (%)	/	0%	0%	0%	0%	0%	0%
